# Supplementary figures and images for: Pbp1 associates with Puf3 and promotes translation of its target mRNAs involved in mitochondrial biogenesis
Source: PLoS Genet. 2023 May 22;19(5):e1010774. doi: 10.1371/journal.pgen.1010774 (PMC10237644; doi:10.1371/journal.pgen.1010774)

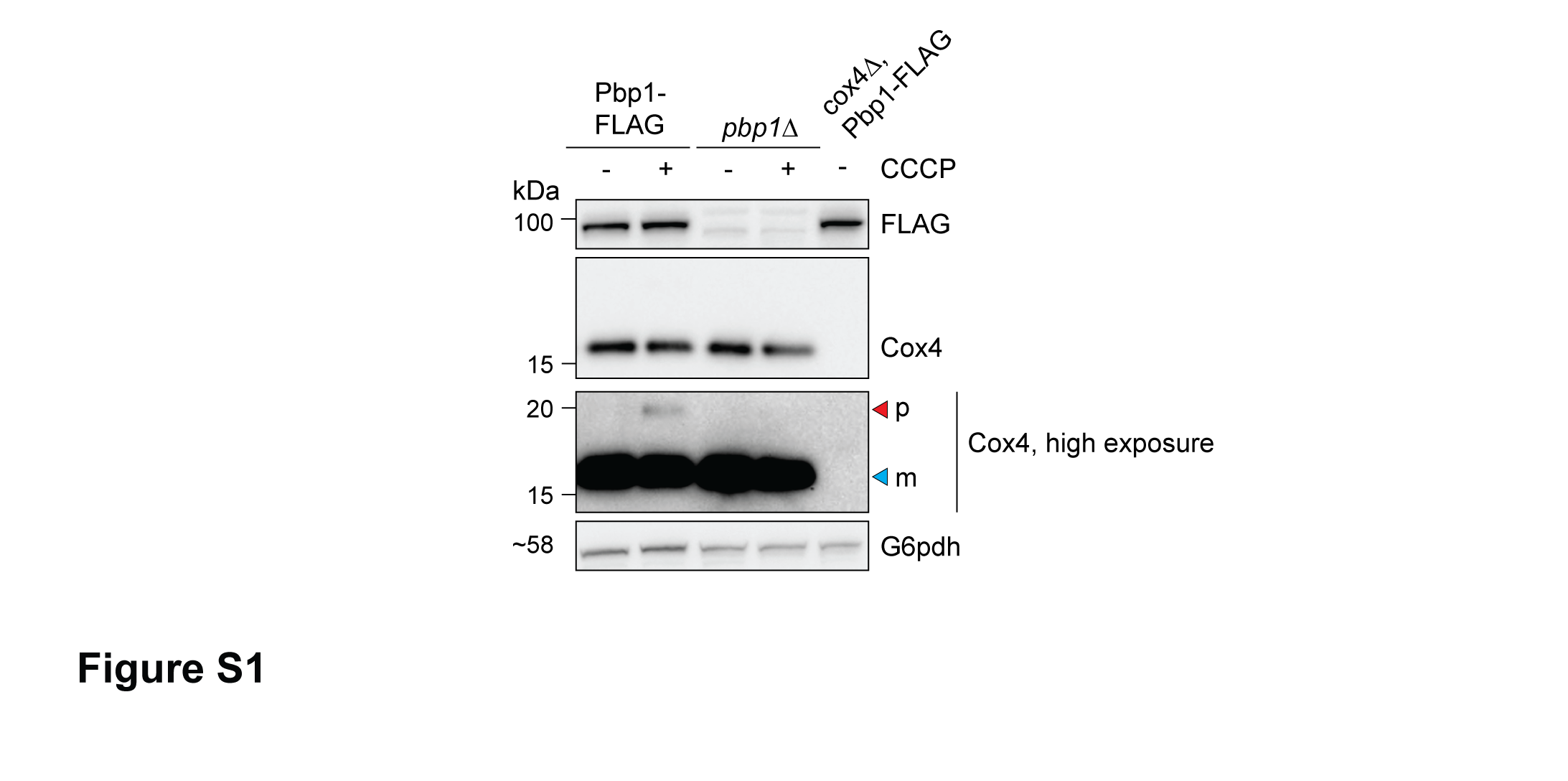

Supplement: S1 Fig — (A) Total protein cell extracts from Pbp1-FLAG or pbp1Δ yeast grown in YPL at 30°C and supplemented with 0.1% DMSO or 40 μM CCCP for 6 h. Pbp1-FLAG, cox4Δ strain serves as control for antibody specificity. p:precursor, m:mature. (TIF) [file pgen.1010774.s001.tif]

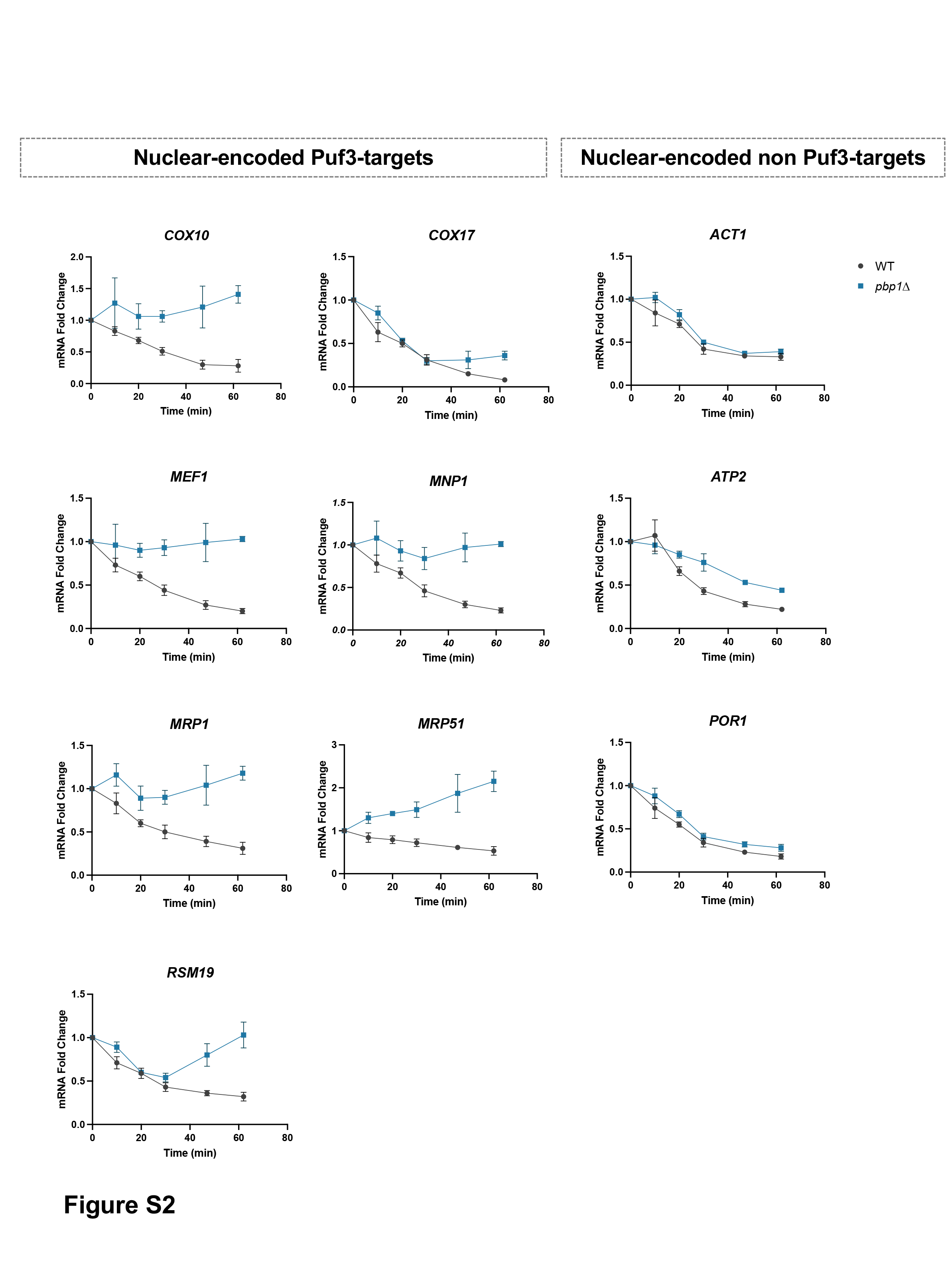

Supplement: S2 Fig — Puf3 target mRNAs in pbp1Δ cells are relatively resistant to decay following treatment with thiolutin. Cells growing at log-phase in YPL media were treated with 3 μg/mL thiolutin and collected at the indicated time-points. The plots represent the fold change in mRNA levels with respect to t = 0 calculated by RT-PCR with normalization to PGK1 mRNA. Error bars represent SD; n = 4. (TIF) [file pgen.1010774.s002.tif]

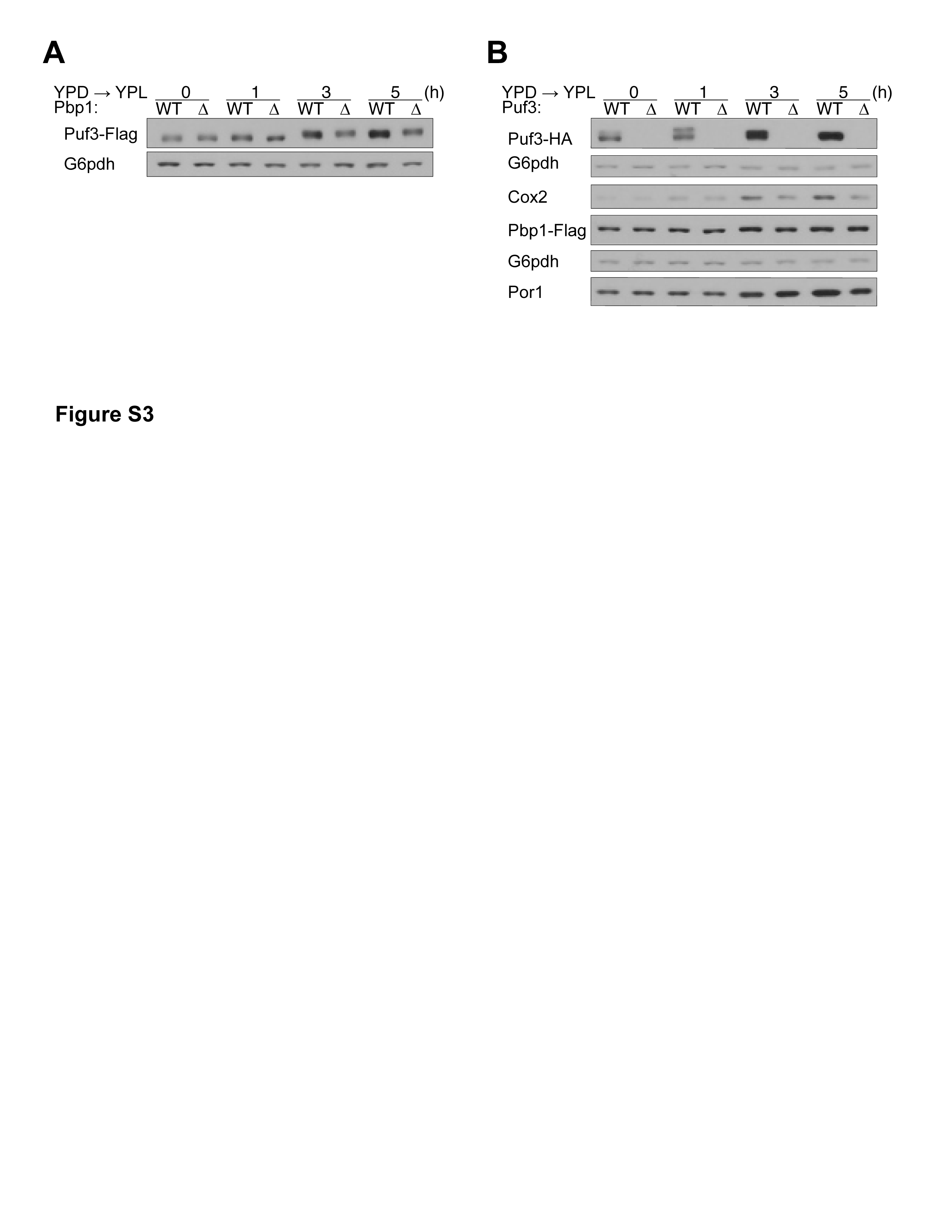

Supplement: S3 Fig — (A) Puf3 protein levels are decreased in cells lacking Pbp1. Cells expressing Puf3-Flag were assayed in the presence or absence of Pbp1 for the indicated proteins by Western blot. (B) Pbp1 protein levels are not affected in cells lacking Puf3. Cells expressing Pbp1-Flag were assayed in the presence or absence of Puf3 for the indicated proteins by Western blot at various time points following switch from YPD to YPL. (TIF) [file pgen.1010774.s003.tif]

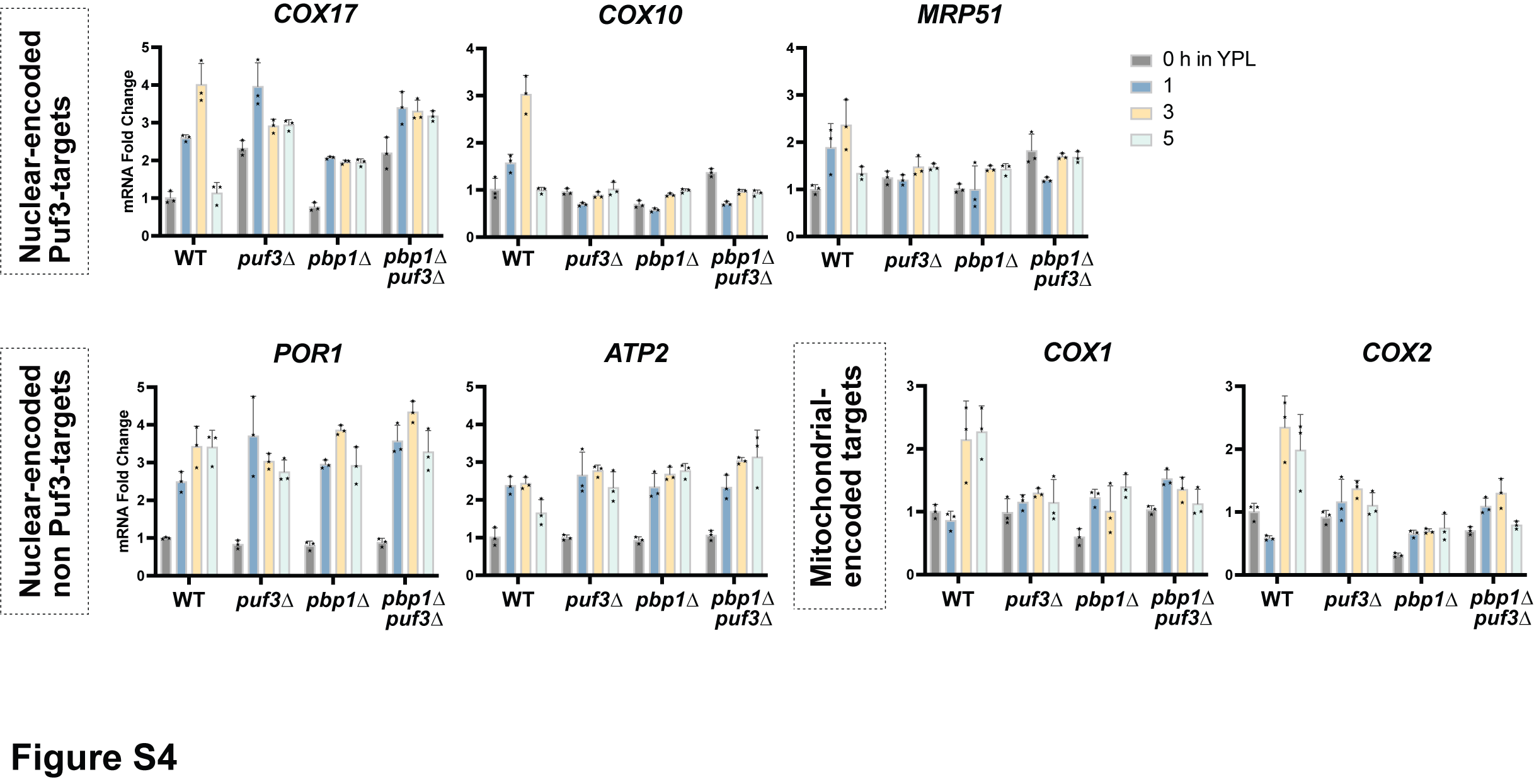

Supplement: S4 Fig — Quantitation of the same panel of transcripts as in Fig 1C in the indicated strains. (TIF) [file pgen.1010774.s004.tif]

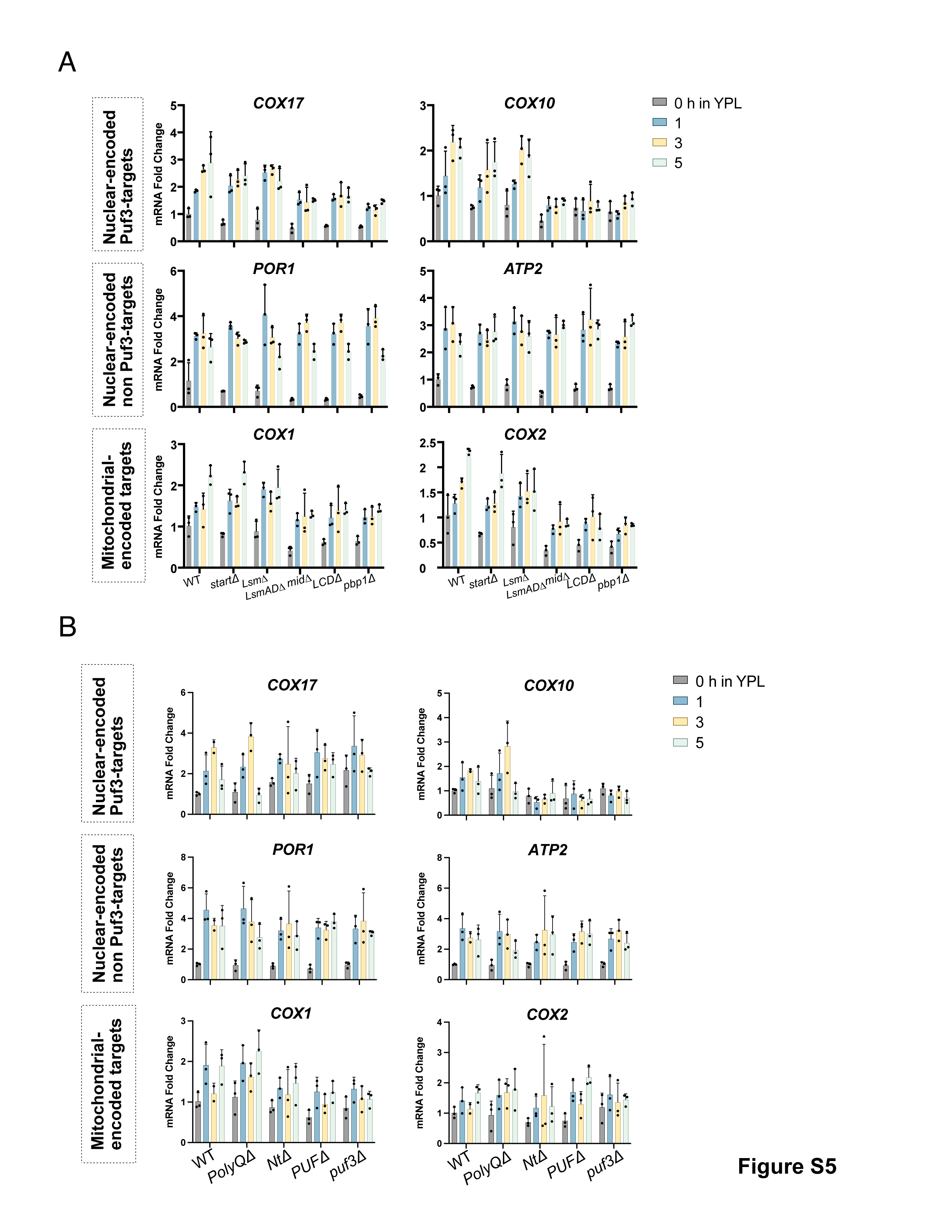

Supplement: S5 Fig — Several mRNAs involved in mitochondrial function were assayed by qRT-PCR in the indicated Pbp1 (A) or Puf3 (B) mutant strains at the indicated time points after switch from YPD to YPL. The abundance of the indicated transcripts was normalized to ACT1. (TIF) [file pgen.1010774.s005.tif]

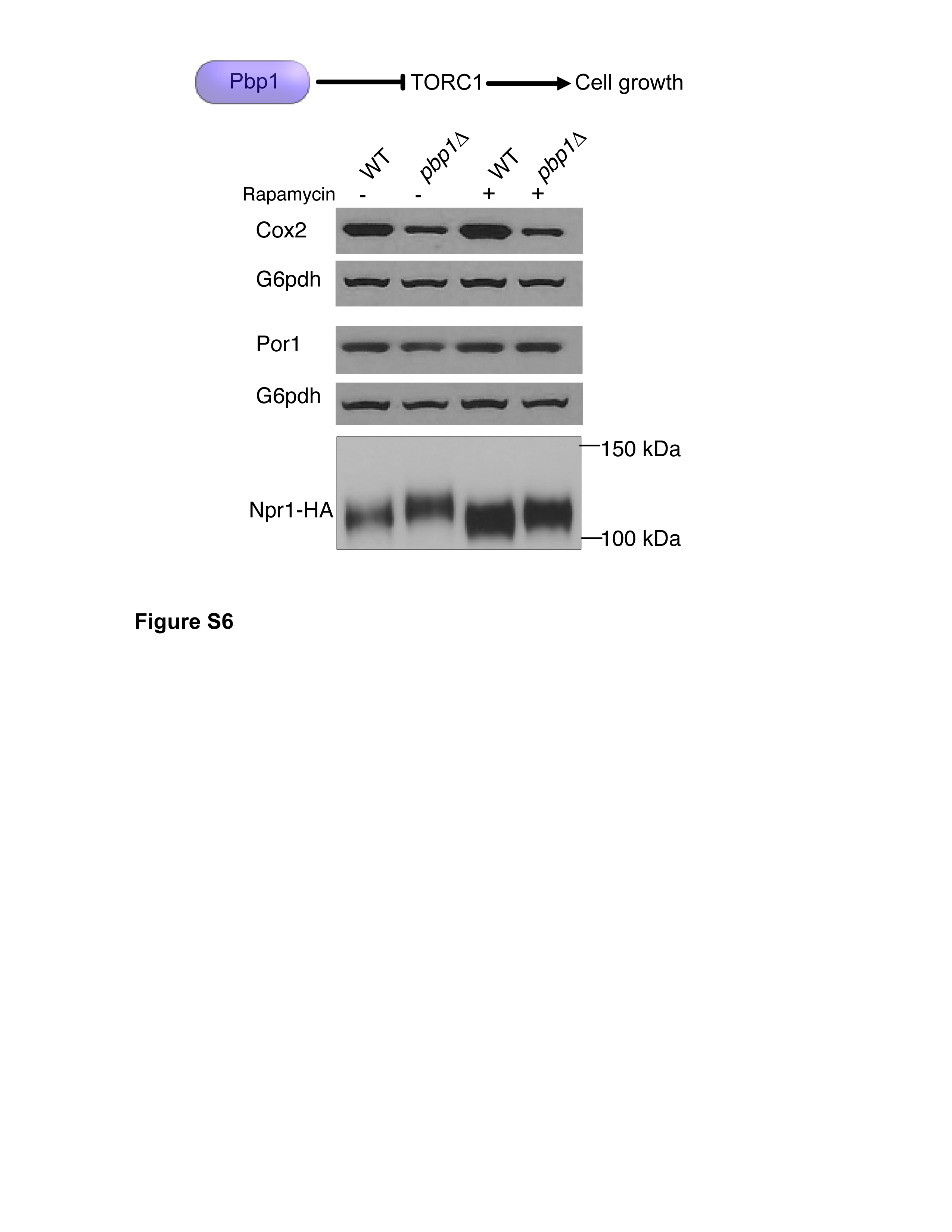

Supplement: S6 Fig — The indicated strains were grown in YPL for 3 h, of which the last 30 min were in the absence (-) or presence (+) of 200 ng/mL rapamycin. Samples were collected, quenched, and extracted for immunoblot analysis of the indicated proteins. Note that rapamycin treatment did not restore protein amounts of Cox2 in pbp1Δ cells. Npr1-HA, a TORC1-dependent substrate, was used as a positive control for rapamycin. (TIF) [file pgen.1010774.s006.tif]
