# Supplementary material for: Pbp1 associates with Puf3 and promotes translation of its target mRNAs involved in mitochondrial biogenesis
Source: PLoS Genet. 2023 May 22;19(5):e1010774. doi: 10.1371/journal.pgen.1010774 (PMC10237644; doi:10.1371/journal.pgen.1010774)
Supplement: S2 Table — (PDF) [file pgen.1010774.s008.pdf]

S2 Table. *Saccharomyces cerevisiae* strains used in this study.

Strain	Genotype	Origin	Strain background
WT	MATa	(41)	CEN.PK
pbp1Δ	pbp1Δ::Hyg	(16)	CEN.PK
puf3Δ	puf3Δ::KanMX6	(9)	CEN.PK
pbp1Δpuf3Δ	pbp1Δ::Hyg, puf3Δ::KanMX6	This paper	CEN.PK
Pbp1-Flag, Puf3-HA	Pbp1-3xFlag::NatNT2, Puf3-HA::KanMX6	This paper	CEN.PK
Puf3-HA	Puf3-HA::KanMX	(9)	CEN.PK
Puf3-Flag, Pbp1-HA	Puf3-3xFlag::NatNT2, Pbp1-HA::Hyg	This paper	CEN.PK
Pbp1-HA	Pbp1-HA::Hyg	(16)	CEN.PK
Pbp1 startΔ-Flag, Puf3-HA	pbp1Δ::pbp1Δa.a.2-49-3xFlag::Hyg, Puf3-HA::KanMX6	This paper	CEN.PK
Pbp1 LsmΔ/LsmADΔ-Flag, Puf3-HA	pbp1Δ::pbp1Δa.a.50-298-3xFlag::Hyg, Puf3-HA::KanMX6	This paper	CEN.PK
Pbp1 midΔ-Flag, Puf3-HA	pbp1Δ::pbp1Δa.a.299-570-3xFlag::Hyg, Puf3-HA::KanMX6	This paper	CEN.PK
Pbp1 LCDΔ-flag, Puf3-HA	pbp1Δ::pbp1Δa.a.571-722-3xFlag::Hyg, Puf3-HA::KanMX6	This paper	CEN.PK
pbp1Δ, puf3-HA	pbp1Δ::Hyg, Puf3-HA::KanMX6	This paper	CEN.PK
Pbp1 M8S-Flag, Puf3-HA	pbp1Δ::pbp1M591, 595, 605, 606, 614, 616, 618, 625S-3xFlag::Hyg, Puf3-HA::KanMX6	This paper	CEN.PK
Pbp1 M8F-Flag, Puf3-HA	pbp1Δ::pbp1M591, 595, 605, 606, 614, 616, 618, 625F-3xFlag::Hyg, Puf3-HA::KanMX6	This paper	CEN.PK
Pbp1 M8Y-Flag, Puf3-HA	pbp1Δ::pbp1M591, 595, 605, 606, 614, 616, 618, 625Y-3xFlag::Hyg, Puf3-HA::KanMX6	This paper	CEN.PK
Puf3 polyQΔ Flag, Pbp1-HA	puf3Δ::puf3Δa.a.398-418-3xFlag::NatNT2, Pbp1-HA::Hyg	This paper	CEN.PK
Puf3 NtΔ Flag, Pbp1-HA	puf3Δ::puf3Δa.a.2-258-3xFlag::NatNT2, Pbp1-HA::Hyg	This paper	CEN.PK
Puf3 PUFΔ Flag, Pbp1-HA	puf3Δ::puf3Δa.a.538-844-3xFlag::NatNT2, Pbp1-HA::Hyg	This paper	CEN.PK
puf3Δ, Pbp1-HA	puf3Δ::Hyg, Pbp1-HA::KanMX6	This paper	CEN.PK
WT, WT reporter	ho::P_{ADH1}-MtRFP P_{ACT1}-GEV P_{GAL1}-cox4(1-21)-yEGFP-mrp51 3'UTR-Hyg	(9)	CEN.PK
WT, mutant reporter	ho::P_{ADH1}-MtRFP P_{ACT1}-GEV P_{GAL1}-cox4(1-21)-yEGFP-mrp51*** 3'UTR-Hyg	(9)	CEN.PK
pbp1Δ WT reporter	ho::P_{ADH1}-MtRFP P_{ACT1}-GEV P_{GAL1}-cox4(1-21)-yEGFP-mrp51 3'UTR-Hyg, pbp1Δ::KanMX6	This paper	CEN.PK
pbp1Δ mutant reporter	ho::P_{ADH1}-MtRFP P_{ACT1}-GEV P_{GAL1}-cox4(1-21)-yEGFP-mrp51*** 3'UTR-Hyg, pbp1Δ::KanMX6	This paper	CEN.PK
Puf3-Flag	Puf3-3xFlag::KanMX6	(9)	CEN.PK
Puf3-Flag, *pbp1Δ*	Puf3-3xFlag::KanMX6, pbp1Δ::Hyg	This paper	CEN.PK
Pbp1-Flag	Pbp1-3xFlag::Hyg	(16)	CEN.PK

Pbp1-Flag, *puf3Δ*	Pbp1-3xFlag::Hyg, puf3Δ::KanMX6	This paper	CEN.PK
Pbp1-Flag, Idh1-GFP, *cox4Δ*	Pbp1-3xFlag::NatNT2, Idh1-GFP::KanMX, cox4Δ::Hyg	This paper	CEN.PK
Npr1-HA	Npr1-HA::KanMX6	This paper	CEN.PK
Npr1-HA, *pbp1Δ*	Npr1-HA::KanMX6, pbp1Δ::Hyg	This paper	CEN.PK

mrp51*** MRP51 3'UTR * denotes one TGTAAATA motif in MRP51 3'UTR has been mutated to ACACAATA
