# Supplementary material for: Pbp1 associates with Puf3 and promotes translation of its target mRNAs involved in mitochondrial biogenesis
Source: PLoS Genet. 2023 May 22;19(5):e1010774. doi: 10.1371/journal.pgen.1010774 (PMC10237644; doi:10.1371/journal.pgen.1010774)
Supplement: S3 Table — (PDF) [file pgen.1010774.s009.pdf]

S3 Table. qRT-PCR primers used in this study.

Primer name	Sequence
Act1-Fw	TCCGGTGATGGTGTTACTCA
Act1-Rv	GGCCAAATCGATTCTCAAAA
GFP-Fw	TCCATGGCCAACCTTAGTCAC
GFP-Rv	CTGGCATGGCAGACTTGAAAA
Cox10-Fw	CTTGTAGACCCGATGGTAAGAAA
Cox10-Rv	GGGCTTGGTCAACTGAAGATA
Atp2-Fw	CCCAGTTGGGAGAGAAACTTTA
Atp2-Rv	CTGCGTGAATTGGCTTTCTTAG
Cox17-Fw	CCAGAAAAGGAGGAGCGGGATA
Cox17-Rv	CGAAGCCATAACCCTTCATGCAC
Cox2-Fw	GTTGATGCTACTCCTGGTAGATT
Cox2-Rv	TGTCCCACACAACTCAGAAC
Por1-Fw	GGCTACAATGAACTGCAAACTAC
Por1-Rv	AATCGGACACCTTAGCCTTAAC
Cox1-Fw	CTACAGATACAGCATTTCCAAGA
Cox1-Rv	GTGCCTGAATAGATGATAATGGT
Mef1_Fw	ACTGATGGTAGCGTTCAATACTC
Mef1_Rv	CACTCTGAATGTAGGGTCTTCC
Rsm19_Fw	ACCGGCAGCTAGACTTTTATC
Rsm19_Rv	ATTGGAGTGCCCTTAGTCATG
Mrp1_Fw	TGGGTCTTTGATGGAGTT
Mrp1_Rv	TTGCGACTAACCACGTAA
Mnp1_Fw	TCGTTCGACACGAAGACTAAGG
Mnp1_Rv	TTGGTGCAGCTTCAACGAAT
Pgk1_Fw	TCATTGGTGGTGGTGACACT
Pgk1_Rv	GCAACACCTGGCAATTC
