# Supplementary material for: Pbp1 associates with Puf3 and promotes translation of its target mRNAs involved in mitochondrial biogenesis
Source: PLoS Genet. 2023 May 22;19(5):e1010774. doi: 10.1371/journal.pgen.1010774 (PMC10237644; doi:10.1371/journal.pgen.1010774)
Supplement: Attachment — Submitted filename: 130335_1_rebuttal_2626532_rrmmy6.docx [file pgen.1010774.s010.docx]

**Manuscript number:** RC-2022-01670

**Corresponding author(s):** Benjamin, Tu

*[Please use this template only if the submitted manuscript should be considered by the affiliate journal as a full revision in response to the points raised by the reviewers.*

*If you wish to submit a preliminary revision with a revision plan, please use our "*[*Revision Plan*](https://docs.google.com/document/u/0/d/1HHp62Tfeb44nd3Gu_3ChB0b8gmMdEog7GjZcZFwHSiQ/edit)*" template.* ***It is important to use the appropriate template to clearly inform the editors of your intentions.****]*

## 1. General Statements [optional]

We thank the reviewers for their time and for the helpful comments. We felt the reviews overall were fair and quite positive. All three reviewers felt the manuscript could be of broad, general interest, especially given the relevance of the protein (Pbp1/ataxin-2) to neurodegenerative conditions and stress granule biology. Reviewer 3 seems to have some doubt there could be specificity for the types of transcripts regulated by Pbp1, given prior studies of mammalian ataxin-2 which implicated 16,000+ mRNAs that could bind via PAR-CLIP experiments! However, our study shows the power of utilizing a simpler model organism and thinking about the metabolic state of cells for elucidating the function of this interesting protein. Although our demonstration of the specificity of Pbp1 for regulating Puf3-target mRNAs involved in mitochondrial biogenesis and mitochondrial function may be surprising to this reviewer, we have the utmost confidence in our data and feel the study represents a highly significant finding that will be of interest to many researchers.

## 2. Point-by-point description of the revisions

*This section is mandatory.* *Please insert a point-by-point reply describing the revisions that were already carried out and included in the transferred manuscript.*

*Reviewer #1 (Evidence, reproducibility and clarity (Required)):*

*In this manuscript, van de Poll et al. aim to further establish the function of poly(A) binding protein-binding protein 1 (Pbp1) and its relationship to the RNA-binding protein Puf3 in regulating the expression of mitochondrial proteins. This work builds upon a solid body of previous studies from this group regarding the function of Puf3 and the role of Pbp1 in regulating TORC1 signaling. Here, the authors show that Pbp1 has a physical and functional interaction with Puf3 and that ablation or disruption of Pbp1 eliminates this interaction and reduces the expression of some Puf3 target proteins. This work provides reliable data supporting a role for Pbp1 in the regulation of mitochondrial protein expression and that there is a clear functional interaction between Puf3 and Pbp1. Nonetheless, there are several issues that should be addressed before the manuscript is suitable for publication.* 

*1.) The model put forward suggests that Pbp1 works to recruit Puf3 to the vicinity of mitochondria, where Puf3 can then promote the expression of its target mRNAs. In Figure 3A, however, deletion of Pbp1 has a stronger affect on the expression of COX2 than deletion of Puf3 alone, which would not be expected if the role of Pbp1 is to modulate Puf3 function. Similarly, the expression of COX2 is higher in the double deletion versus the Pbp1 single deletion. The authors should attempt to clarify this experimentally, or at least make mention of alternative mechanisms for Pbp1 which may be causing this.*

We have mentioned alternative mechanisms in the text. We suggest that Puf3-target mRNAs also can be translated through Tom20, in the absence of Puf3 (p. 8, ref 18). In single deletion strains lacking Pbp1 (but with Puf3 present), Puf3 may direct some of its target mRNAs to decay pathways, leading to lower Cox2 expression compared to double deletion strains that also lack Puf3. We performed qPCR analysis of several Puf3-target and other mRNAs in pbp1∆puf3∆ double deletion strains and some transcripts (e.g., COX17) would support this possibility (Fig S4).

*2.) The authors mention that the increased pull-down of Puf3 with Pbp1 in respiratory conditions suggests that the Pbp1-Puf3 interaction is responsive to the cellular metabolic state (Figure 3B). However, the increase in Puf3 expression makes it difficult to compare the interactions between the two conditions.*

Yes this is correct, we stated this in the legend of Fig 4B as: “Increased amounts of Puf3 are associated with Pbp1 in respiratory conditions.” We clarified in the text that Puf3 expression increases in respiratory conditions and this likely explains the increased pull-down of Puf3 with Pbp1 (p. 10).

*3.) The authors only look at a very small subset of Puf3 target mRNAs using qPCR when it would be much more informative and overall convincing to examine a larger amount using RNA-seq experiments.*

We conducted an RNA-seq experiment to compare transcriptomes of WT vs pbp1∆ cells in fermentative (YPD) vs respiratory (YPL) conditions and observed mRNAs with functions associated with mito-translation and mito-respiration (i.e., Puf3-targets) to be most differentially expressed – and majority of these are lower in abundance in pbp1∆ cells (new Fig 2).

*4.) The authors consistently mention that Pbp1 function is helping to stabilize Puf3 target mRNAs. However, if the authors wish to prove this particular mode-of-action, more direct evidence should be provided, such as a pulse-chase experiment. Otherwise, other models allowing for increased mRNA abundance should be noted.*

Using thiolutin which is the standard for such expts, we measured mRNA half-lives of several Puf3-target and other mRNAs (COX17, COX10, POR1, ACT1) by qPCR in WT vs pbp1∆ cells. However, these data turned out to be difficult to interpret, as several “control” mRNAs exhibited different decay profiles in pbp1∆ vs WT cells, and their behavior was different in respiratory conditions compared to what was reported in common glucose media. Nonetheless, the data are included as Fig S2, and the important observation is that each of the Puf3-target mRNAs tested behaves similarly following thiolutin treatment, compared to non Puf3-target mRNAs. Given that Puf3-target mRNAs were more stable in pbp1∆ cells (compared to PGK1) following thiolutin treatment, we have deleted the term “stabilize” throughout the text. The exact fate of these mRNAs in normal vs pbp1∆ mutant cells will require more sophisticated investigation in future studies.

*5.) Given the proposed model, one would expect Puf3 to have reduced mRNA binding upon deletion of Pbp1. It would be interesting to examine Puf3 mRNA binding, perhaps through cross-linking immunoprecipitation (CLIP), to see if this indeed is the case. This would provide further direct evidence that Pbp1 is functioning through Puf3 and facilitating its function. Similarly, the authors mention that Pbp1 contains putative RNA binding domains, however, they make no mention if these domains may contribute to its function in mitochondrial protein expression.* 

We performed an RNA-IP experiment to test whether Puf3 has reduced binding to its target mRNAs in the absence of Pbp1. In new Fig 7, Puf3 is still able to bind its mRNA targets in the absence of Pbp1. However, the association of Pbp1 with these mRNAs is reduced in puf3∆ knockouts. Such results are perhaps expected as Puf3 has been shown to bind in a sequence-specific manner to a ~8 nt motif in the 3’UTR of its target mRNAs. However, it is unclear whether the Lsm / LsmAD domains of Pbp1 actually bind RNAs directly (hence our use of the term “putative”) - they may be involved in protein-protein interactions. Moreover, Fig 5 shows that deletion of both domains has no apparent effect on mitochondrial protein expression. We prefer to address the role of the Lsm and LsmAD domains of Pbp1 in a future study.

*Reviewer #1 (Significance (Required)): 

Overall, this manuscript provides a modest advance, but one that could prove to have important implications for the field-especially if the Pbp1 findings prove relevant to its human ortholog, ataxin-2. The advance is limited by the robustness of the specific molecular model proposed and the extent to which the Pbp1-Puf3 relationship is examined on the gene-expression level. 


Reviewer #2 (Evidence, reproducibility and clarity (Required)): 

In this manuscript, the authors found that the pbp1∆ mutant grew poorly on nonfermentable carbon source medium (YP Lactate medium) and that the pbp1∆ mutant had decreased amount of Cox2 protein, a cytochrome c oxidase. The pbp1∆ mutant also had decreased amounts of COX17, COX10, and MRP51 mRNAs. Since these mRNAs are target mRNAs of the RNA-binding protein Puf3, the authors next investigated the relationship between Pbp1 and Puf3. The analysis of the GFP reporter gene containing Puf3-binding sites in the 3' UTR showed that the levels of GFP reporter mRNA and protein were decreased in the pbp1∆ mutant strain. This reduction was dependent on the Puf3-binding site in the 3' UTR. Next, the authors examined the genetic interaction between the pbp1∆ and puf3∆ mutants, and found that the levels of Cox2 protein were reduced in the two mutants. Finally, the authors showed the interaction between Pbp1 and Puf3 by co-immunoprecipitation and determined the regions of Pbp1 and Puf3 required for the interaction. They also showed that these regions in Pbp1 and Puf3 proteins are also important for the regulation of Cox2 protein levels. The story is very clear and the data is reliable. However, the data from Western blotting should be shown quantitatively to make the results more reliable. Also, although the story is based on the reduction of Cox2 protein level, it would be better to discuss whether other proteins or mRNAs should be considered as well. 

Major comments: 

Figure 2C. 
Protein levels of GFP should be quantified and the data should be shown.*

These data have been quantified (now Fig 3C).

*Figure 3. 
Not only the Cox2 protein level but also mRNA levels of COX2, COX17, COX10, MRP51, etc in pbp1∆ mutant, puf3∆ mutant and pbp1∆ puf3∆ double mutant should be shown. Then the point of action of Pbp1 and Puf3 would become clearer.*

The mRNA levels have been determined by qPCR and are now in Fig S4.

*Figure 4. 
For the domain analysis of Pbp1 protein, showing differences in cell proliferation as in Figure 1B would indicate the physiological importance of the domain.*

Growth curves of the various Pbp1 domain deletions have been performed and shown in Fig 5D. They do support the physiological importance of the domain(s).

*Figure 4B. 
Quantification of the amount of co-immunoprecipitated proteins would indicate the strength of binding.*

These data have been quantified (now Fig 5B).

*Figure 4C. 
Protein levels should be quantified and the data presented.* 

These data have been quantified (now Fig 5C).

*Line 153-8 
The description of Line 153-8 is not appropriate for this position because it breaks up the flow of the story before and after.* 

These text have been moved as requested.

*Minor comments: 

Line 153 
Isn't the following the first reference cited for Pbp1 is a negative regulator of TORC1? 
Transient sequestration of TORC1 into stress granules during heat stress 
Terunao Takahara 1, Tatsuya Maeda 
Mol Cell. 2012 Jul 27;47(2):242-52. doi: 10.1016/j.molcel.2012.05.019. Epub 2012 Jun 21. 

Ref19. 
Ref 19 also shows that the pbp1∆ mutant strains grow poorly on the medium containing glycerol and lactate as carbon sources. 

Overall, the gene is not italicized.* 

These requested edits to the references and text have been made in the revised version of the manuscript.

*Reviewer #2 (Significance (Required)): 

This manuscript analyzes the relationship between Pbp1 and Puf3 in yeast. Since these proteins are evolutionarily conserved from yeast to humans and are also associated with disease in humans, this reviewer believes this manuscript will be of interest to a wide audience.* 

*Reviewer #3 (Evidence, reproducibility and clarity (Required)): 

In this study, van de Poll et al. describe the involvement of a cytosolic RNA-binding protein (Pbp1) in the transcriptional regulation of mitochondrial biogenesis during the shift from fermentative to respiratory growth in budding yeast. Using the advantage of yeast genetics and molecular biology, they show that a number of mitochondrial transcripts and proteins are downregulated in pbp1Δ cells both under normal growth conditions and during respiratory shift. Since Puf3, another RNA-binding protein, is known to regulate the fate of these transcripts, they further investigate the interaction between Pbp1 and Puf3. Through a series of biochemical assays, they characterize the interaction between Pbp1 and Puf3 taking place through their low-complexity domains, which is suggested by authors to stabilize and promote the translation of Puf3-target transcripts. 

As stated in the manuscript, Pbp1 is an evolutionarily conserved protein, encoded by ATXN2 gene in humans. Due to its involvement in multiple neurological disorders, it has been widely studied in mouse models and patient samples. The transcriptome profiles of Atxn2-KO mouse liver and cerebellum have been published (albeit having used a relatively older microarray technology for today's standards), revealing a prominent dysregulation of global translational machinery, and the ER-protein secretion pathway (Fittschen et al., 2015). These findings were followed by numerous studies showing dysregulations of distinct transcript pools under examination. Moreover, a PAR-CLIP study showed ATXN2 to associate with ~16.000 transcripts, 8000 of which depended on its interaction with PABPC1 (Yokoshi et al., 2014). In that study, ATXN2 was shown to preferentially bind to target transcripts on 3'-UTR AU-rich sequences. In addition to PABPC1, some other RNA-binding proteins, like TDP-43, were also shown to modulate the indirect interaction of ATXN2 with transcripts, while it also maintained direct interactions through its Lsm and LsmAD domains. Altogether, the mammalian data on ATXN2 thus far depicts it as an avid interactor of numerous RNA-binding proteins and countless transcripts, including microRNAs. The authors however have not cited or compared their findings with this vast array of mammalian literature (with the exception of two properly discussed papers in Discussion). 

Considering its stress-responsive nature and association with RNP granules, it is plausible to assume that ATXN2/Pbp1 could regulate certain groups of transcripts in terms of their stability (in stress granules and p-bodies) or active translation under given environmental conditions and cellular state. The work of van de Poll et al. in this regard is an important step in expanding our knowledge about the many downstream effects of ATXN2/Pbp1. Yet, the following issues should be solved: 

1) The pre-eminence of the proposed group of affected transcripts (i.e. those associated with mitochondrial biogenesis) has to be empirically established. Among all its interactions with other RNA-binding proteins, how important or dominant is the interaction of Pbp1 with Puf3 for mitochondrial biogenesis during the shift towards respiratory growth? Line 74 in the Results says "Analysis of a panel of nuclear- and mitochondrial-encoded mRNAs..."; how broad was this panel? how were the genes selected? Considering the fact that ATXN2/Pbp1 is associated with an immense number of transcripts, hand-picking a number of Puf3 targets and selectively analyzing their expression will surely give some significant dysregulations. Therefore, an unbiased transcriptomic survey is necessary to see all Pbp1-dependent dysregulations during respiratory shift. Since it's a process that requires heavy mitogenesis, one can assume that many dysregulations will concern mitochondrial factors. Indeed, proteome surveys in Atxn2-KO mouse tissues and pbp1Δ yeast (under fermentative growth and stress) point out to a strong mitochondrial dysregulation, so it raises hopes to see an even stronger Pbp1 impact during the respiratory shift in yeast. Then, bioinformatic analyses can reveal what proportion of those are Puf3 targets. If the authors' premise is valid, then the Puf3-targets will stand out in the transcriptome data, and give them an unbiased, solid and much stronger base for the following interaction analyses. They can then compare this dataset to the readily available mouse transcriptome from Atxn2-KO or polyQ-disease models, and strengthen their hypothesis about ATXN2/Pbp1 regulating mitochondrial biogenesis in association with Puf3. 

The Results text about Figure 1 in its current state is an overstatement of the available data. Line 81-84 suggests mitochondrially encoded Cox2 levels are reduced because some mitoribosome subunits are Puf3 targets, but pbp1Δ itself is known to have altered mitochondrial membrane potential which negatively impacts mitochondrial import. So, the reduction of Cox2 levels (and potentially other mitochondrial-encoded proteins, it was never checked) may have nothing to do with Puf3, but rather be a direct consequence of reduced mitoribosome import in pbp1Δ. In order to make this statement, the total mitochondrial translation rates of WT and pbp1Δ strains would have to be compared, and if they are found the same, a selective effect on Puf3-target proteins has to be shown among many tested candidates. Same applies to line 86 "the specific decrease in Puf3-target mRNAs in pbp1Δ cells" referring to Figure 3C. This statement cannot be made without analyzing a larger group of transcripts including the targets of other RNA-binding proteins. The current data does not support any specific dysregulation of Puf3-targets, it just shows some Puf3 targets to be dysregulated, however without the knowledge of how many significant among all Puf3-targets, or how significant are Puf3-targets compared to others. An unbiased, high-throughput transcriptome data, and detailed bioinformatic analyses should replace Figure 1C. The high variation among replicates at 1h-3h-5h time points is also alarming, and puts the reproducibility of these experiments under question.*

As mentioned in the response to Reviewer 1, we performed an unbiased RNA-seq experiment comparing transcriptomes of WT vs pbp1∆ cells. The data are quite striking and strongly support our hypothesis (new Fig 2). To address the reviewer’s concern that pbp1∆ phenotypes may be due to altered mito membrane potential and mito protein import, we have performed an experiment to examine import of Cox4, which is a protein substrate that is commonly used for this purpose (note COX4 is not a Puf3-target mRNA). Steady-state Cox4 protein amounts are similar in WT vs pbp1∆ cells. Moreover, following treatment of cells with the uncoupler CCCP, there is more of the “pre”-processed Cox4 form in WT cells compared to pbp1∆ cells. These data would argue against the reviewer’s hypothesis and are included in the revised manuscript as Fig S1. Since every mitochondrial ribosomal subunit gene transcript is a target of Puf3 (PMID: 16254148) and therefore subject to regulation by Pbp1, we would argue that a defect in mito biogenesis (due to compromised translation of these mRNAs) precedes and may explain any subsequent defect in mito membrane potential.  *2) Stabilization of mRNAs 
The basal reduction in the mRNA levels of the reporter construct in pbp1Δ is a strong but not necessarily direct evidence of stabilization by Pbp1. mRNA half-life analyses (i.e. degradation curves) should be performed with desired targets to measure stability in WT and pbp1Δ strains.*

As mentioned above in the response to Reviewer 1, we performed mRNA half-life analyses for several transcripts in WT vs pbp1∆ strains using thiolutin treatment. The results are not straightforward to interpret as loss of Pbp1 led to a stabilization of Puf3-target mRNAs (relative to PGK1), however all Puf3-target mRNAs that we examined exhibited similar decay profiles. Thus, we deleted the term “stabilize” and further determination of the fate of these mRNAs in the absence of new transcription will require more careful and sophisticated experiments. *3) Cox2 levels 
Regarding Line 125: "Cox2 protein levels, which are dependent on the translation of Puf3-target mRNAs": This may be generally true, but the data here suggests otherwise. pbp1Δ cells have completely diminished Cox2 levels, whereas puf3Δ have approx. 50% reduction. This means that Pbp1 is more important to maintain normal Cox2 levels, and does so independent of Puf3. In contrast, puf3Δ "rescues" some of the defect in pbp1Δ cells and increases Cox2 abundance to ~50%. As stated above, Cox2 reduction in pbp1Δ could be a direct consequence of mitochondrial membrane depolarization unrelated to Puf3, and could be accompanied by many other non-Puf3-targets being downregulated. Therefore, the authors should refrain from "Cox2 levels are dependent on the translation of Puf3-target mRNAs" statements throughout the text without an experimental proof in the context of pbp1Δ strain.*

See response to Reviewer 1. We believe that in the absence of Pbp1, Puf3 may now preferentially promote decay of various mito biogenesis transcripts, leading to apparently lower Cox2 levels. Moreover, per the results of our Cox4 experiment (Fig S1), we would respectfully disagree with the reviewer’s hypothesis. Nonetheless, we included an additional statement that mitochondrial membrane depolarization, as a consequence of reduced expression of numerous Puf3-targets, could also contribute to lower Cox2 abundance (p. 6 and 15).  *4) Pbp1-Puf3 interaction 
The authors state that Pbp1-Puf3 interaction is required for Puf3-target mRNA stabilization and translation. This suggests that Pbp1 stabilizes this pool of mRNAs because of its interaction with Puf3 primarily, not the mRNAs themselves. One general question while studying the interaction between two RNA-binding proteins is whether they interact in an RNA-dependent manner in vivo. The co-IP analyses show the interaction between Pbp1 and Puf3 to increase under respiratory shift as expected. However, in co-IPs from cell lysates, many RNA-binding proteins may seemingly interact due to their association with translation machinery at that given time. But this does not mean "direct" protein-protein interaction, just a co-existence around actively translating ribosomes. In order to ensure the direct interaction of these two proteins, the same co-IPs should be performed with/out RNase treatment of the lysate (many protocols available online). Only if Pbp1-Puf3 interaction persists in RNase+ samples, they can conclude a direct interaction. In addition, RNA-immunoprecipitation analyses should be performed in Pbp1-Flag and Pbp1-Flag/ puf3Δ strains to check if the association of Pbp1 with Puf3-target mRNAs indeed depends on Puf3.*

This is a good suggestion. We performed an experiment to test whether RNase treatment alters interaction between Pbp1 and Puf3 and there was minimal effect, supporting the hypothesis that the interaction may be direct. We also performed RNA-IP of Pbp1 in the presence of absence of Puf3 (new Fig 7). As the reviewer predicted, the RNA-IP enrichment of Puf3-target mRNAs was reduced in puf3∆ strains, suggesting that the association of Pbp1 with such transcripts depends on Puf3. It is known from work by others that Puf3 contains a PUF domain that enables sequence-specific binding to a motif in the 3’UTR of its target mRNAs, so these results are quite sensible.

*Minor comments: 
• Second sentence of the Abstract ("How mutations in its mammalian ortholog ataxin-2 are linked to neurodegenerative conditions remains unclear") is semantically incorrect. The term "linked" suggests an observed but uncharacterized effect of a genetic variation on a certain syndrome. Diseases can be linked to a chromosome or a locus without knowing the exact causative mutation. How the CAG repeat expansion mutations in ATXN2 are causative of SCA2, ALS or Parkinson-plus syndromes are very well known. One should also be careful with using "mutations" as a general term in the context of ATXN2, because there are certain variations in and around ATXN2 locus leading to a decrease in its activity and metabolic problems, which is far from its neurodegeneration-causing mutations. If the authors meant to state that the pathological mechanism is unclear by this sentence, that would also be a negligence of the extensive literature around this topic. Multiple disease models in mouse, Drosophila and C. elegans collectively point out to an RNA metabolism deficit, caused by toxic ATXN2 aggregates that sequestrate other RNA-binding proteins and their target transcripts. The specific downstream effects involve synaptic strength, Calcium-related action potentials, ER stress and cholesterol/sphingolipid synthesis. Therefore, this sentence could be rephrased to "PolyQ expansion mutations in its mammalian ortholog ataxin-2 lead to spinocerebellar dysfunction due to toxic protein aggregation." and simply avoid going into mechanistic details as it is not necessary for this manuscript. 

• Lines 241-243: "Human sequencing" is also an incorrect term. Can be rephrased to "PolyQ expansion mutations in ATXN2 are associated with SCA2 and ALS". The references 22-24 are the first association of polyQ mutations with SCA2, however a reference for ALS is missing. Elden et al. Nature 2010 should be cited here. 

• The authors should discuss the relevance of these findings to the mammalian ortholog of Pum3, namely PUM1/2. Afterall, it is also a very important conserved protein and well-studied in mammalian literature.*

These changes to the text and references have been made.

*• Following a better characterization of the transcript pools selectively affected by Pbp1 (meaning a transcriptome survey), a graphical abstract sort of scheme could be useful in putting the findings in perspective and conveying the message.* 

We decided not to include a graphical abstract at this time, since it is difficult for us to “picture” what is going on inside an Pbp1-containing RNP granule at this time.

*Reviewer #3 (Significance (Required)): 

The intricate experiments characterizing the nature of interaction between Pbp1 and Puf3 (Figures 3B, 4, 5, 6) are quite convincing. However, some fundamental questions remain especially regarding the primary rationale of studying Pbp1-Puf3 relationship and the breadth of some conclusions. 
The data are of general interest to a broad audience. 
The statistical tests in Figure 1 are of concern. 
The reviewer(s) has experience both with yeast molecular biology and with mammalian Atxn2 function.*
